# Behavior of *Yersinia enterocolitica* in Foods

**DOI:** 10.4061/2011/420732

**Published:** 2011-10-23

**Authors:** Md. Latiful Bari, M. Anwar Hossain, Kenji Isshiki, Dike Ukuku

**Affiliations:** ^1^Food Analysis Research Laboratory Center for Advanced Research in Sciences, University of Dhaka, Dhaka-1000, Bangladesh; ^2^Department of Microbiology, University of Dhaka, Dhaka-1000, Bangladesh; ^3^Division of Marine Life Science, Research Faculty of Fisheries Science, Hokkaido University, 3-1-1, Minato-cho, Hakodate, Hokkaido 041-8611, Japan; ^4^Food Safety Intervention Technologies, Eastern Regional Research Center, USDA, 600 East Mermaid Lane, Wyndmoor, PA 19038, USA

## Abstract

*Yersinia enterocolitica* are ubiquitous, being isolated frequently from soil, water, animals, and a variety of foods. They comprise a biochemically heterogeneous group that can survive and grow at refrigeration temperatures. The ability to propagate at refrigeration temperatures is of considerable significance in food hygiene. Virulent strains of *Yersinia* invade mammalian cells such as HeLa cells in tissue culture. Two chromosomal genes, inv and ail, were identified for cell invasion of mammalian. The pathogen can cause diarrhoea, appendicitis and post-infection arthritis may occur in a small proportion of cases. The most common transmission route of pathogenic *Y. enterocolitica* is thought to be fecal-oral via contaminated food. Direct person-to-person contact is rare. Occasionally, pathogenic *Y. enterocolitica* has been detected in vegetables and environmental water; thus, vegetables and untreated water are also potential sources of human yersiniosis. However, the isolation rates of pathogenic *Y. enterocolitica* have been low, which may be due to the limited sensitivity of the detection methods. To identify other possible transmission vehicles, different food items should be studied more extensively. Many factors related to the epidemiology of *Y. enterocolitica*, such as sources, transmission routes, and predominating genotypes remain obscure because of the low sensitivity of detection methods.

## 1. Introduction

*Yersinia enterocolitica* is a psychotropic zoonotic pathogen which causes acute gastroenteritis [1] and occasionally more serious disease in humans. In some countries it rivals *Salmonella* as a foodborne pathogen, and, because it can grow at refrigeration temperature [2], it is an increasing concern in terms of food safety. Infection with *Y. enterocolitica* can cause a variety of symptoms depending on the age of the person infected. Infection with *Y. enterocolitica* occurs most often in young children under 5 years old [3]. Most cases of yersiniosis occur sporadically in children [4]. The predominant symptoms in humans, particularly in young children, are fever, abdominal pain, and diarrhea, which is often bloody [5]. In older children and adults, the consequences of yersiniosis are severe and include acute infections, pseudoappendicitis, and extraintestinal long-term sequelae such as reactive arthritis and erythema nodosum [6, 7]. Secondary immunological sequelae, such as reactive arthritis, are not uncommon, especially in HLA-B27-positive individuals. 

*Yersinia enterocolitica* is thought to be a significant food-borne pathogen, even though pathogenic strains have seldom been isolated from foods. Pigs are assumed to be the main reservoir of pathogenic *Y*.* enterocolitica* because pig is so far the only animal species from which pathogenic strains have frequently been isolated [8]. Several domestic animals like dogs, cats, cows, sheep, and horses and several wild [9] animals like rodents (mainly mice), monkeys, deer, and foxes have also been incriminated as potential reservoirs [10]. 

The geographical distribution of *Y. enterocolitica *is diverse. *Y. enterocolitica *has more than 50 distinct serotypes (on the basis of antigenic variations in cell wall lipopolysaccharide), and few of them are pathogenic. O:8 is the primary infectious serotype in the USA followed by O:3, O:5,27, O:13a, 13b, O:20, O:9, and so forth, [11, 12]. Serotype O:3 is the most frequently isolated type in humans in Europe [3]. In China, serotype O:3 is primarily found in infections followed by O:9, and O:8 [13]. Furthermore, various serotypes demonstrate geographical specificity; for example, the predominant serotype in Australia, Europe, and Canada is O:3 [14], O:8 in Japan [15] and O:9 in Scandinavia, The Netherlands [16].

The emergence of yersiniosis is probably also related to changes that have occurred in livestock farming, food technology, and the food industry. Of greatest importance are changes in the meat industry, where meat production has shifted from small-scale slaughterhouses, with limited distribution patterns, to large facilities that process thousands of pigs each day and distribute their products nationally and internationally. Farm sizes have increased, and animal husbandry methods have also become more intensive. While many modern slaughter techniques reduce the risk of meat contamination, opportunities for animal-to-animal transmission of the organism and for cross-contamination of carcasses and meat products exist on a scale that was not known a few decades ago. In addition, advances in packaging and refrigeration now allow industry and consumers to store foods for much longer periods, a significant factor with regard to a cold-adapted pathogen such as *Y. enterocolitica. *In studying raw pork, higher detection rates have been obtained by PCR targeting chromosomally encoded several virulence genes than by culture methods [3]. In some case-controlled studies, an increased risk of yersiniosis has been demonstrated when raw or undercooked pork was consumed [14]. Nevertheless, the epidemiology of *Y. enterocolitica* infections is complex and remains poorly understood [3].

## 2. *Yersinia enterocolitica* Infection

Although *Y*. *enterocolitica* is a frequent and important cause of human disease in temperate zones, *Y*. *enterocolitica* infections have also been sporadically reported in tropical areas like China [19] and Japan [15]. The organism has been isolated from many foods, but foodborne outbreaks are rare, and most infections are sporadic. There have been relatively few foodborne outbreaks attributed to *Y. enterocolitica *in developed countries, for example, Japan, and The Netherlands [15, 16], as well as in developing countries, for example, Bangladesh and Iraq [20, 21].

*Y. enterocolitica* can cause gastrointestinal symptoms ranging from mild self-limiting diarrhoea to acute mesenteric lymphadenitis, which can lead to appendicitis [3]. The clinical manifestations of the infection depend to some extent on the age and physical state of the patient, the presence of any underlying medical conditions, and the bioserotype of the organism. Gastroenteritis, caused by *Y. enterocolitica*, is the most frequent form of yersiniosis, typically affecting infants and young children under 5 years [5]. In older children and young adults, acute yersiniosis can present as pseudoappendicular syndrome, which is frequently confused with appendicitis. Sometimes extra-intestinal long-term sequelae, including reactive arthritis, erythema nodosum, uveitis, glomerulonephritis, and myocarditis have been reported. Postinfection manifestations are mainly seen in young adults [3]. Sepsis is a rare complication of *Y. enterocolitica* infection, except in patients who have a predisposing underlying disease or are in an iron-overloaded state. Sepsis can also occur during blood transfusion [22]. In most cases, the infection is self-limiting, and no antimicrobial therapy is needed. However, in severe cases, antimicrobials may be useful. Antimicrobial resistance among human *Y*. *enterocolitica* strains has shown to be low, but multiresistant strains have also been reported [3], and, thus, antimicrobial therapy should always be based on the results of sensitivity tests.

*Yersinia enterocolitica *has evolved into an apparently heterogeneous collection of organisms encompassing six biotypes differentiated by physiochemical and biochemical tests (1A, 1B, 2, 3, 4, and 5) (Table 1) and more than 50 serotypes differentiated by antigenic variation in cell wall lipopolysaccharide. Of the six biotypes, biotype 1A is the most heterogeneous and encompasses a wide range of serotypes (Table 2), of which serotypes O:5, O:6,30, O:6,31, O:7,8, O:10, as well as O-nontypable strains are isolated most often [17]. The virulence of the pathogenic biotypes, namely, 1B and 2–5, is attributed to the presence of a highly conserved 70-kb virulence plasmid, termed pYV/pCD and certain chromosomal genes [23]. The biotype 1A strains of *Y. enterocolitica*, on the other hand, have been reported to lack pYV plasmid which encodes virulence factors including *Yersinia * adhesin A (YadA) and Ysc-Yop type III secretion system (TTSS) as well as chromosomally borne virulence genes including *ail*, *myfA*, *ystA*, *ysa*, and the high pathogenicity island- (HPI-) associated iron acquisition system [24]. 

**Figure 1:**
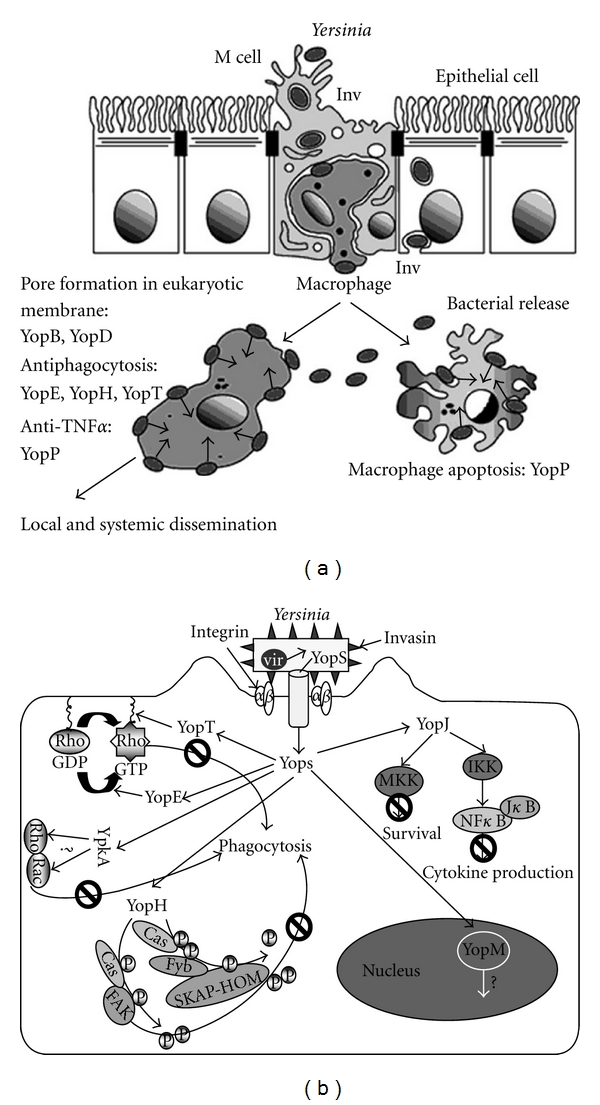
Physiopathological infection of *Yersinia *(adopted from [27]).

*Y. enterocolitica* infection is typically initiated by ingestion of contaminated food or water. *Yersinia enterocolitica *(Figure 1) usually causes a diarrhoeal disease, whereas *Y. pseudotuberculosis *causes mild enteric symptoms that may be followed by mesenteric lymphadenitis and sometimes systemic diffusion. Yersiniae cross the intestinal epithelium primarily through the FAE, in the Peyer's patches of the ileum [25]. Invasin (Inv), a 103 kDa outer membrane protein of *Y. pseudotuberculosis* binds b1 integrins that are also expressed apically on M cells. Inv-negative mutants still adhere to and invade M cells, but at a much lower level than the wild-type strain, and their colonisation potential for Peyer's patches is considerably reduced [26].

Other *Yersinia *surface proteins such as Ail, PsaA, and YadA may account for residual invasion of inv mutants [28]. Once the dome is reached, yersiniae survive attack by resident macrophages by expressing an antiphagocytic strategy caused by the injection, through a plasmid-encoded type III secreton, of three protein effectors, YopH, T, and E, which disrupt cytoskeletal assembly [29]. YopH, a tyrosine phosphatase, dephosphorylates paxillin, p130cas, and FAK that are involved in the assembly of cytoskeletal complexes required for phagocytosis [30]. YopT provokes the depolymerisation of actin filaments by inducing redistribution of the RhoA GTPase [31]. YopE expresses a GAP function that inhibits the small GTPases of the Rho family involved in phagocytosis [32]. Yersiniae, therefore, remain essentially extracellular in infected Peyer's patches and mesenteric lymph nodes. This allows their extracellular survival and possible Inv-mediated entry into epithelial cells [27]. 

*Y. enterocolitica* strains belonging to certain few bioserotypes can cause human disease. Most strains associated with yersiniosis belong to the following bioserotypes: 1B/O:8; 2/O:5,27; 2/O:9; 3/O:3; 4/O:3. These bioserotypes have been shown to have different geographical distributions. Strains largely responsible for human yersiniosis in Europe, Japan, Canada, and the USA belong to the bioserotype 4/O:3 [33]. Strains of five biotypes (1B, 2, 3, 4, and 5) can carry the pYV, which is required for full expression of virulence, and several chromosomally encoded virulence determinants. Strains of biotype 1A lack the virulence-associated markers of pYV-bearing strains and are considered to be nonpathogenic. However, growing clinical, epidemiological, and experimental evidence suggests that some biotype 1A strains are virulent and can cause gastrointestinal disease [17]. Several studies have been conducted to investigate the distribution of different virulence genes (*ail*, *inv*, *yst*, *yadA*, *virF,* and *yopT*) among *Y. enterocolitica* strains by PCR [33]. Pathogenesis of *Y*. *enterocolitica *is mediated by virulence factors encoded on chromosomes and plasmids [34]. A correlation between biotypes and the presence of plasmid and chromosomal virulence genes has been found. However, plasmid-borne genes (*yadA*, *virF*, and *yopT*) have been detected with variable efficiency owing to heterogeneity within the bacterial population for the presence of the virulence plasmid.

## 3. Epidemiology of *Y. enterocolitica*

Indirect evidence suggests that food, particularly pork, is an important link between the pig reservoir and human infections. In case-controlled studies, a correlation has been demonstrated between the consumption of raw or undercooked pork and the prevalence of yersiniosis [35, 36]. To identify reservoirs of infections, transmission vehicles, and associations between clinical cases, several DNA-based methods have been used to subtype *Y. enterocolitica* strains (Table 3). However, the high genetic similarity between *Y. enterocolitica* strains and the predominating genotypes among the strains have limited the benefit of these methods in epidemiological studies. Thus, many factors related to the epidemiology of *Y. enterocolitic*a, such as sources and transmission routes of yersiniosis, remain obscure.

## 4. Reservoirs

The evidence is not yet complete as to whether humans serve as reservoirs of *Y. enterocolitica*. It is isolated from low percentage of asymptomatic humans. However, it appears that the animal kingdom is a significant reservoir. Some members of the animal kingdom harbor unique serotypes of *Y. enterocolitica *which have not been implicated in human infections. Animals have long been suspected of being reservoirs for *Y. enterocolitica* and, hence, sources of human infections [11]. Numerous studies have been carried out to isolate *Y. enterocolitica* strains from a variety of animals. However, most of the strains isolated from animal sources differ both biochemically and serologically from strains isolated from humans with yersiniosis [10]. The pigs have been implicated as a major reservoir of *Y. enterocolitica *serotypes involved in human infections although a definite connection between the isolation of *Y. enterocolitica *from the pigs and human illness remains to be established. The incidence of *Y. enterocolitica *in pigs varies not only from country to country but also within a country. *Y. enterocolitica* strains that belong to bioserotypes associated with human disease have frequently been isolated from tonsils, tongues, and faecal samples of slaughtered pigs [14]. The rate of isolation of *Y. enterocolitica *from tonsils and tongues of pigs is generally greater than the rate of isolation from feces or fecal materials. In several countries, *Y. enterocolitica* of bioserotype 4/O:3 has been shown to be the predominant bioserotype in asymptomatic pigs. *Y. enterocolitica *serotype O:3 has been almost exclusively isolated from pigs in some European countries, like Denmark, Belgium, Finland, Germany, Sweden, and Switzerland [8, 37–40]. A lower prevalence has been reported in Italy, Greece, and Poland (Table 4)  [41–43]. Some investigators concluded that the O:3 strain is a normal inhabitant of the oral cavity of pigs and also involved in human infection.

Examination of the throat flora from pigs in Ontario for *Y. enterocolitica *found the incidence of serotype O:3 to vary from 20% for tonsils to 50% for throat swabs and 55% for tongues. In contrast, there were no isolations of serotype O:3 from throat swabs taken from pigs in the western provinces of Canada. This incidence of serotype O:3 in pigs correlates well with the human incidence of the same serotype which is 81% for all human isolations of *Y. enterocolitica *in the eastern provinces and 4% in the western provinces of Canada. The opposite relationship is true for serotype O:5,27. The majority of O:3 and O:5,27 were positive for autoagglutination, a test which has been associated with virulence. The results suggest that pigs are an important source of human infections with both O:3 and O:5,27 [44]. 

In Guangxi, Mainland China, *Y. enterocolitica *were isolated from 48.4% of the pigs with diarrhea, and most of the isolates were O:3 with two isolates belonged to serotype O:9 [45]. These two serotypes are considered to be pathogenic for humans. 

In another study in China, *Y. enterocolitica *(1,295 strains) was isolated from diarrhea patients, livestock, poultry, wild animals, insect vectors, food, and the environment. They were studied for epidemiology distribution using bacterial biochemical metabolism tests, their virulence genes, and pulsed-field gel electrophoresis (PFGE) subtyping. The data showed that 416 of the 1,295 strains were pathogenic, where the pathogenic Chinese isolates were of serotypes O:3 and O:9. These two serotypes were found in livestock and poultry, with pigs serving as the major reservoir. The geographic distribution of pathogenic isolates was significantly different, where most of the strains were isolated from the cold northern areas, whereas some serotype O:3 strains were recovered from the warm southern areas. By the analysis of the data of the Ningxia Hui Autonomous Region, the phenomenon of “concentric circle distribution” was found around animal reservoirs and human habitation. The clustering of PFGE showed that the patterns of the pathogenic strains isolated from diarrhea patients were identical compared to those from the animals in the same area, thus, suggesting that the human infection originated from the animals [19]. 

In many years of surveillance in China for *Y. enterocolitica*, no pathogenic O:8 strains have been found where the isolated O:8 serotypes lacked the major virulence genes, and, in contrast to the O:3 and O:9 strains, none of the O:8 isolates were from humans. These O:8 isolates lack *ail*, *ystA*, *yadA*, and *virF *genes but possess the *ystB *gene, and all belong to biotype 1A. These O:8 strains did not kill mice and could protect immunized mice against challenge with a pathogenic O:8 strain. Compared to the Chinese pathogenic O:3 and O:9 strains which have similar pulsed-field gel electrophoresis patterns, the 39 Chinese O:8 animal and food isolates were different from the pathogenic O:8 reference strains. This suggests the O:8 strains lacking virulence determinants may not disseminate rapidly in humans and are maintained in animal reservoirs, and, therefore, exhibit higher variance and divergence from the virulent type [13]. 

Sixteen different isolates of *Y. enterocolitica *were recovered from porcine tongues, including six O:8, four O:6,30, two O:3, and one each of O:13,7, O:18, and O:46 [46]. All the serotype O:8 isolates were virulent to mice, causing the death of adults after oral challenge [46]. 

In a cross-sectional study, individual pigs on eight swine operations were sampled for the presence of *Y. enterocolitica*. On each farm, both feces and oropharyngeal swabs were collected from pigs in five different production phases: gestating, farrowing, suckling, nursery, and finishing. A pig was considered positive if either sample tested positive. Of the 2,349 pigs sampled, 120 (5.1%) tested positive, and, of those, 51 were *ail* positive (42.5% of *Y. enterocolitica *isolates). On all farms, there was a trend of increasing prevalence as pigs mature. Less than 1% of suckling piglets tested positive for *Y. enterocolitica*. Only 1.4% (44.4% of which were *ail* positive) of nursery pigs tested positive, but 10.7% (48.1% of which were *ail* positive) of finishing pigs harbored *Y. enterocolitica*. Interestingly, gestating sows had the second highest prevalence of *Y. enterocolitica *at 9.1% (26.7% of which were *ail *positive), yet *Y. enterocolitica *was never detected from the farrowing sows [47].

Occasionally, pathogenic *Y. enterocolitica* strains, mostly of bioserotype 4/O:3, have been isolated from domestic animals like dogs, cows, horses, sheep, and cats [38]. Dogs excrete this organism in feces for several weeks after infection. *Y. enterocolitica *or related species were isolated from 50% of cows in Scotland, and the isolates varied in serotypes [48]. *Y. enterocolitica* strains of biotypes 2 and 3 and serotypes O:5,27 and O:9 have sporadically been isolated from slaughter pigs, cows, sheep, and goats; however, the reservoir of these bioserotypes is not clearly established [49–51]. Thus, pets may be one source of human infections because of their close contact with people, especially young children. *Y. enterocolitica *were isolated from wild animals [52, 53] for example, from 16 of 495 small wild animals (mainly mice) and from 1 of 38 foxes [52], the isolated serotype were O:6, O:5A, O:4, and O:9. Wild rodents and pigs have been shown to be reservoirs for *Y. enterocolitica* O:8 strains in Japan [9]. Strains of very rare bioserotypes, such as bioserotype 5/O:2, 3, have been isolated from sheep, hares, and goats and bioserotype 3/O:1, 2a, 3 from chinchillas.

All environmental isolates, except one, had a *NotI* profile identical to that of an isolate recovered in pig feces from the same farm. This suggests that the environment represents a source of contamination of pigs by *Y. enterocolitica*. However, because the prevalence of pathogenic *Y. enterocolitica* in the environment was clearly lower than that in pigs, the pigs probably are the main source of pathogenic isolates on the farms. Several studies using different typing methods have been conducted to compare human strains with animal, mostly pig, strains. Most of the reports support the hypothesis that pigs are the main source of human *Y. enterocolitica* infections [33].

## 5. Contamination of Food and Environment

Food has often been suggested to be the main source of *Y. enterocolitica* infection, although pathogenic isolates have seldom been recovered from food samples. Raw pork products have been widely investigated because of the association between *Y. enterocolitica* and pigs. However, the isolation rates of pathogenic bioserotypes of *Y. enterocolitica* have been low in raw pork, except for edible pig offal, with the most common type isolated being bioserotype 4/O:3. The low isolation rates of pathogenic *Y. enterocolitica* in food samples may be due to limited sensitivity of culture methods [39]. The occurrence of pathogenic *Y. enterocolitica* in some foods has been estimated by different detection methods. In all of these studies, the prevalence was higher by PCR than by the culturing method.

Prevalence of *yadA*-positive *Y. enterocolitica* in food has been studied in Finland [39]. The highest detection rate was obtained from pig offal, including pig tongues (83%), livers (73%), hearts (71%), and kidneys (67%). The detection rate was higher in minced meat with the PCR method than with the culture method (Table 5). Thisted Lambertz and Danielsson-Tham [54] detected *ail*-positive *Y. enterocolitica* in 10% (9/91) of raw pork samples (loin, fillet, chop, ham, and minced meat) and in one of 27 ready-to-eat pork products. Surprisingly, Vishnubhatla et al. [55] found a high occurrence of *yst*-positive *Y. enterocolitica* in ground beef. In the same study, *yst*-positive *Y. enterocolitica* was also detected in tofu by real-time PCR. These PCR results indicate that the true rate of contamination of pathogenic *Y. enterocolitica* in pork and other processed meats and foods is underestimated using culture methods.

*Y. enterocolitica *has been isolated from raw milk in many countries, like Australia, Canada, Czechoslovakia, and USA. There were also a few reports on the isolation of this pathogen from pasteurized milk. It may be due to the malfunction in the pasteurization process leading to inadequate treatment or postprocess contamination, or it may be due to the contamination of heat-resistant strains of *Y. enterocolitica*. However, heat-resistant strains have not been reported. 

Stern, 1982, reported that *Y. enterocolitica *could grow in whole milk at 3°C. Also the reduction of psychrotrophic bacteria in milk after pasteurization would enable a poor competitor and opportunistic pathogen such as *Y. enterocolitica *to grow better in pasteurized than in raw milk. So, the presence of this pathogen in pasteurized milk should be a cause for concern. *Y. enterocolitica *was isolated from 9.2% of cheese curd samples in Canada [60].

*Y. enterocolitica *are commonly detected in meat and poultry products. The level of this pathogen was found consistently in high numbers on vacuum-packed meats with a pH above 6 held at low temperature [60]. Growth of this pathogen is enhanced in cooked meats or at low temperature whereas competitive microorganisms are inactivated. 

Prevalence of pathogenic *Y. enterocolitica *in different sources in Bavaria is presented. The highest isolation rate of pathogenic *Y. enterocolitica *(67%) was found in tonsils of slaughter pigs. No pathogenic strains were isolated from cattle, sheep, turkey, and horses. *ail*-positive *Y. enterocolitica *was detected in dogs (5%), cats (3%), and rodents (3%) by real-time PCR. Pathogenic *Y. enterocolitica *was isolated only from raw pork, especially from edible offal (51%). All pathogenic *Y. enterocolitica *isolates from nonhuman sources were belonging to bioserotype 4/O:3. All *Y. enterocolitica *4/O:3 strains were susceptible to most of the tested antibacterial agents [61]. 

Strains of *Y. enterocolitica *have been isolated from oysters, mussels, shrimp, blue crab, fish, chicken salad, stewed mushrooms, cabbage, celery, and carrots [60]. 

No pathogenic *Y. enterocolitica* has been detected in fish and chicken samples in Finland; however, three (3%) lettuce samples were positive. In Korea, Lee et al. [62] isolated one *ail*-positive *Y. enterocolitica* strain of bioserotype 3/O:3 from 673 samples of ready-to-eat vegetables, which supports that vegetables can be a source of human infection. Furthermore, Sakai et al. [15] reported a foodborne outbreak of *Y. enterocolitica* O:8 in Japan where the same PFGE pattern was obtained from all patient and salad isolates. Recently, *Y. enterocolitica* 2/O:9 has been isolated from chicken eggshell surfaces in Argentina [63]. Using PFGE, *XbaI* patterns revealed a genomic heterogeneity among the strains, which suggests different contamination sources. Contamination of the egg surface might have occurred from contact with other *Y. enterocolitica*-contaminated animal products, such as pork, during collection on farms or during transportation or handling in retail shops.

In a case-controlled study, untreated drinking water has been reported to be a risk factor for sporadic *Y. enterocolitica* infections in Norway [36]. Drinking water has been relatively widely investigated and revealed to be a significant reservoir for nonpathogenic *Y. enterocolitica*. However, Sandery et al. [58] detected pathogenic *Y. enterocolitica* in 10% of environmental water, and Fãlcao et al. [64] recently tested 67 *Y. enterocolitica* strains isolated in Brazil from untreated water for the presence of virulence genes. They found that all 38 strains of serotype O:5,27 possessed *inv*, *ail,* and *yst *genes, suggesting that water may be responsible for human infection with *Y. enterocolitica*. In Japan, the *Y. enterocolitica* O:8 strains have been isolated from stream water [9, 65]. Distribution of genotypes of *Y. enterocolitica* 4/O:3 strains in butcher shops in Munich has been studied with PFGE using NotI, ApaI, and XhoI enzymes [66]. Twelve genotypes were obtained among 33 isolates from 14 pork and two environmental samples, demonstrating that several different strains were distributed in butcher shops. The genotypes differed among butcher shops, possibly because raw material was purchased from different sources. In most shops, more than one genotype was found, indicating that the raw material was contaminated with different strains. These results show that pathogenic *Y. enterocolitica* can easily be transmitted from slaughterhouses via contaminated raw material to the retail level.

## 6. Possible Routes of Transmission

The most common transmission route of pathogenic *Y. enterocolitica* is thought to be fecal-oral via contaminated food. Direct person-to-person contact is rare. Lee et al. [67] reported *Y. enterocolitica* O:3 infections in infants who were probably exposed to infection by their careers. This may happen when basic hygiene and hand-washing habits are inadequate. In July 2006, person-to-person transmission was observed in a familial outbreak of *Y. enterocolitica *bioserotype 2/O:9 in Japan [68]. The possible source of this infection was an infected carrier suffered from diarrhea [68]. In addition, the outbreak of diarrheal disease due to *Yersinia enterocolitica *bioserotype 1/0:5 was reported in hospitalized patients, which was the indication of a nosocomial outbreak due to *Yersinia enterocolitica* [69]. Indirect person-to-person transmission has apparently occurred in several instances by transfusion of contaminated blood products [22]. One transmission link may be direct contact with pigs, a common risk for pig farmers and slaughterhouse workers. However, transmission of pathogenic *Y. enterocolitica* from pigs to humans has not yet been proven. 

The main sources of human infection are assumed to be pork and pork products. Pathogenic *Y. enterocolitica* can be transmitted from slaughterhouses to meat-processing plants and then to retail level via contaminated pig carcasses and offal [66, 70]. Contaminated pork and offal are important transmission vehicles from retail shops to humans [70]. Cross-contamination of offal and pork will occur directly or indirectly via equipment, air and food handlers in slaughterhouses [59], retail shops [66], and residential kitchens. The detection rate of pathogenic *Y. enterocolitica* in raw pork products has been shown to be high. However, consumption of raw pork would play only a limited role in the development of yersiniosis as this is not a common habit in most developed countries. Nevertheless, in Germany, raw minced pork with pepper and onion is a delicacy that can be purchased in ready-to-eat form from butcher shops. Transmission probably more often occurs via cooked pork and other food products that have been undercooked or improperly handled. 

Pet animals have also been suspected as being sources of human yersiniosis because of their close contact with humans, especially young children [34]. However, transmission from pets to humans has not yet been proven. Pathogenic *Y. enterocolitica* may be transmitted to humans indirectly from pork and offal via dogs and cats [38]. Transmission of *Y. enterocolitica* 4/O:3 to pets via contaminated pork has been studied using PFGE with NotI, ApaI, and XhoI enzymes. A total of 132 isolates, of which 16 were from cat and dog faeces and 116 from raw pork samples, were studied in Finland. The predominant genotype recovered from pig heart, liver, kidney, tongue, and ear samples was also found in the cat, whose diet consisted mostly of raw pig hearts and kidneys. The dog, which was fed raw minced pork, excreted the same genotype found in the minced meat. These results show that raw pork should not be given to pets because pathogenic isolates can easily be transmitted from highly contaminated raw pork to pets. Dogs and cats may be an important transmission link of pathogenic *Y. enterocolitica* between pigs and young children [34].

## 7. Factors Influencing Survival and Growth

*Y. enterocolitica* is facultative organism and is able to multiply in both aerobic and anaerobic conditions.

### 7.1. Temperature

The ability of *Y. enterocolitica* to multiply at low temperatures is of considerable concern to food producers. The reported growth range is −2 to 42°C [71]. Optimum temperature is 28-29°C [72]. *Y. enterocolitica* can multiply in food such as meat and milk at temperatures approaching and even below 0°C [73]. It is important to recognize the rate at which *Y. enterocolitica* can multiply, which is considerably greater than that of *L. monocytogenes* [74]. Results showed that, in a food with a neutral pH stored at 5°C, *Y. enterocolitica* counts may increase from 10/mL to 2.8 × 10^7^/mL in 5 days. Toxin production by this pathogen is affected by growth temperature and the composition of food items. Toxigenic *Y. enterocolitica *produced heat-stable enterotoxin in milk at 25°C but not at 4°C [75]. Strains which grew well at 4°C in milk did not produce significant amount of toxin to be detected by infant mouse assay [76]. Most *Y. enterocolitica *cells will be killed or injured when being stored during frozen storage at −20°C. When ground beef inoculated with *Y. enterocolitica *was stored at −20°C for 30 days, approximately 83% of the inoculated cells were destroyed and 24% of the survivors were sublethally injured [60]. 

### 7.2. pH

The minimum pH for growth has been reported between 4.2 and 4.4 [77], while in a medium in which the pH had been adjusted with HCl, growth occurred at pH 4.18 and 22°C [78]. The presence of organic acids will reduce the ability of *Y. enterocolitica* to multiply at low pH. Acetic acid is more inhibitory per gram molc than lactic and citric acid at a given pH [78]. Bactericidal activity order is acetic acid > lactic acid > citric acid > sulphuric acid. Bhaduri [79] performed an experiment by changing the pH of the food items at pH 4, 5, and 6. Number of viable cells decreased but 95% of the surviving cells retained the virulence plasmid with their virulence characteristics. However, plasmid-containing cells did not survive at pH 3 [79].

### 7.3. Water Activity

The minimum water activity at which growth occurred is 0.96. This bacterium was able to grow in 5% salt, but not in 7% salt. Stern et al. [77] tested four strains of *Y. enterocolitica* and reported that 0.945 Aw and 7% salt was bactericidal to all 4 strains tested, when incubated at 3°C, but at 25°C both bactericidal and bacteriostatic effects were observed. At 9% NaCl and 25°C, all 4 strains were killed. Bhaduri et al. [79] performed an experiment by changing the salt concentration of the food items to 0.5, 2, and 5%. Number of viable cells decreased, but 96% of the surviving cells retained the virulence plasmid with their virulence characteristics, indicating that there was no effect of NaCl (0.5, 2.0, and 5.0%) on pYV stability [79].

### 7.4. Preservatives/Disinfectants

The growth of *Y. entericolitica* is retarded by potassium sorbate up to 5000 ppm at pH 6.5 in a dose-dependent manner. At pH 5.5 concentrations above 1000 ppm virtually eliminate growth or cause inactivation depending on the dose. Sodium nitrite at a concentration of 150 ppm retarded growth on bologna. Treatments with ozone (1.4 and 1.9 ppm) and with ozonated water (1 min exposure) reduce pathogen loading [80]. Modified atmosphere packaging at 100% N_2_ and CO_2_/N_2_ gas mixers inhibited the growth of *Y. entericolitica* at refrigeration temperatures.

## 8. Growth and Survival in Foods

The ability to propagate at refrigeration temperature in vacuum-packed foods with a prolonged shelf-life is of considerable significance in food hygiene. *Y. enterocolitica* may survive in frozen foods for long periods [81]. *Y. enterocolitica* is not able to grow at pH < 4.2 or >9.0 [82] or salt concentration greater than 7% (Aw < 0.945) [83].* Y. enterocolitica *is not heat-resistant bacteria; with D value at 62.8°C for 15 enterotoxigenic and 6 nonenterotoxigenic cultures ranged from 0.7 to 17.8 sec. in sterile whole milk, the heat-treated cells were counted on trypticase soy agar with yeast extract [75]; it indicates that it does not survive pasteurization. The organism does not survive pasteurization or normal cooking, boiling, backing, and frying temperatures. Heat treatment of milk and meat products at 60°C for 1–3 min effectively inactivates *Y. enterocolitica *[73]. D values determined in scalding water were 96, 27, and 11 seconds at 58, 60, and 62°C, respectively. In another report [84], three raw milk isolates of *Y. enterocolitica *had D values at 62.8°C from 0.24 to 0.96 min in sterile whole milk. However, if the initial level of *Y. enterocolitica *is very high, complete destruction may not occur during pasteurization [60]. Sublethal injury of *Y. enterocolitica *may occur when the cells are treated at 47°C for 12–70 min [60]. 

 A comparison of published and predicted generation times for *Y. enterocolitica* in raw pork at 7°C, 0.5% NaCl (w/v), and pH 5.5–6.5 shows GTs of 8.4–12.4 hours (published) and 8.15–5.05 hours (predicted). However, according to many reports, the ability of *Y. enterocolitica* to compete with other psychrotrophic organisms normally present in food may be poor [85]. In contrast, a number of studies have shown that *Y. enterocolitica* is able to multiply in foods kept under chill storage and might even compete successfully [86, 87]. The effect of lactic acid (concentration range 1.0 to 1.1% v/v within a pH range of 3.9 to 5.8 at 4°C) on growth of *Y. enterocolitica* O:9 is greater under anaerobic than aerobic conditions, although the bacterium has proved to be more tolerant of low-pH conditions under anaerobic atmosphere in the absence of lactic acid [88].

Pig carcasses are often held in chilling rooms for 2–4 days after slaughter prior to cutting. Prepackaged raw meat products may remain in retail chill cabinets for more than a week, depending on the product, packaging, package atmosphere, and rate of turnover. Pathogenic strains of *Y. enterocolitica* might propagate considerably during the course of this relatively long storage period.

As a facultative organism, the growth of *Y. enterocolitica* is drastically affected by a gaseous atmosphere. Under anaerobic conditions, *Y. enterocolitica* is unable to grow in beef at pH 5.4–5.8, whereas growth occurs at pH 6.0 [89]. One hundred percent CO_2_ is reported to inhibit the growth of *Y. enterocolitica* [89]. In the study of Gill and Reichel [71], *Y. enterocolitica* was inoculated into high beef DFD (dark-firm-dry) meat. Samples were packaged under vacuum or in oxygen-free CO_2_ atmosphere maintained at atmospheric pressure after the meat had been saturated with gas and stored at −2, 0, 2, 5, or 10°C. In vacuum packs, *Y. enterocolitica* grew at all storage temperatures at rates similar or faster than those of the spoilage microflora. In CO_2_ packs, the bacterium grew at both 5 and 10°C, but not at lower temperatures. Growth of *Y. enterocolitica* was nearly totally inhibited both at 4 and 10°C in a 60% CO_2_/0.4% CO mixture, while the bacterial numbers in samples packed in high O_2_ mixture (70% O_2_/30% CO_2_) increased from about 5 × 10^2^ bacteria/g at day 0 to about 10^4^ at day 5 at 4°C and to 10^5^ at 10°C. Growth in chub packs (stuffed in plastic castings) was even higher [89]. 

The influence on *Y. enterocolitica *counts of a gradual increase of carbon dioxide concentrations (percentage by volume in air) during packaging and storage of ground pork meat artificially contaminated with this pathogen was evaluated. Ground meat was packaged under customary conditions using modified atmospheres with various carbon dioxide percentages (0, 30, 50, 70, and 100% CO_2_ by volume; for atmospheres of less than 100% CO_2_, the rest of the gas was O_2_). The packs were stored at 2°C for 12 days. *Y. enterocolitica *counts were not significantly different (*P* > or =0.05) in the ground pork packaged under the various CO_2_-enriched atmospheres. The growth of *Y. enterocolitica *was nearly entirely inhibited in all tested modified atmospheres containing the protective CO_2_. However, in ground pork packaged with 100% oxygen, there was a significant decrease (*P* < or =0.05) for *Y. enterocolitica *from 4.30 log CFU/g (day 0) to 3.09 log CFU/g at the end of the storage time (day 12). The decrease was presumably due to the marked increase in aerobic plate count seen only in those packages stored under 100% O_2_. Packaging with high CO_2_ concentrations had significant inhibitory effect (*P* < or =0.05) on the growth of mesophilic aerobic bacteria [90]. 

Mohammad and Draughon [91] investigated the growth characteristics of *Y. enterocolitica* strains in pasteurized milk at 4°C. Pasteurized milk was inoculated with 10 or 1000 cells/mL of *Y. enterocolitica*. *Y. enterocolitica* competed well with the background microflora and reached levels of log 5.0 to 7.0/mL after 7 days. However, a study by Stern et al. [77] indicated that while *Y. enterocolitica* has the capacity for growth in milk at refrigeration temperatures, it is a poor competitor with common spoilage organisms.

Some strains of *Y. enterocolitica* are even able to grow in water at low temperatures (4°C) [92]. In a study, autoclaved tap water (pH 6.5) was chlorinated according to conventional water treatment practices, resulting in a free residual level of approximately 0.05 mg/L after contact time of 30 min. A 3.0 log reduction for *Y. enterocolitica* and *E. coli* exposed to 0.2 mg/L Cl_2_ was obtained in 20–180 and 20–25 sec, respectively, depending on the bacterial strain, plasmid content (*Y. enterocolitica* O:3 harbouring a 40–50 MDa virulence plasmid exhibits enhanced resistance to chlorine), and temperature [93].

Bansal et al. [94] performed an experiment to determine whether the presence of pYV plasmid affects the susceptibility of *Y*. *enterocolitica *to widely used antimicrobial agents like chlorine and heavy metals. According to them plasmid-bearing (pYV+) *Y*. *enterocolitica* was less susceptible to the antimicrobial action of chlorine and heavy metals compared with the isogenic plasmidless (pYV−) derivative. This difference was, however, observed only with bacteria cultured at 25°C but when cells cultured at 37°C were also found to be less susceptible to the antimicrobial action of these agents. These results indicate that the susceptibility of *Y*. *enterocolitica* to these agents was influenced both by the presence of the virulence plasmid and the temperature at which the cells were cultured [94].

Experiments were conducted to determine the effectiveness of oregano and nutmeg essential oils (EOs) on the growth and survival of *Y. enterocolitica *and *Listeria monocytogenes *in broth culture and in Iranian barbecued chicken. Ready-to-cook Iranian barbecued chicken was prepared according to the common practice with 1, 2, and 3 microL/g of oregano and nutmeg EOs. The test and control (without EOs) samples were inoculated with *Y. enterocolitica *to a final concentration of 6 to 7 log CFU/g and stored at 3, 8, and 20°C. Microorganisms were counted just before and at 24, 48, and 72 h after storage. However, the oregano EO had a greater effect on *Y. enterocolitica *(MIC = 0.16 microl/mL) than did the nutmeg EO (MIC = 0.25 microl/mL). In ready-to-cook Iranian barbecued chicken, the log CFU per gram of the bacteria after up to 72 h of incubation was not decreased significantly by various combinations of oregano and nutmeg EOs (1, 2, and 3 microl/g) and storage temperatures (3, 8, and 20°C) when compared with control samples (without EOs). Although examination of spices in culture media can yield accurate microbiological data, without complementary tests in foods, these data are of limited value for assessing food safety [95].

## 9. Conclusion

*Yersinia enterocolitica* is an important zoonotic pathogen that can cause yersiniosis in humans and animals. Pigs are assumed to be the main source of human yersiniosis, even though a definite connection between pathogenic *Y. enterocolitica* strains isolated from pigs and human infections has not been established. A close genetic relationship between pig and human strains of *Y. enterocolitica* has been demonstrated by several DNA-based methods. However, the high similarity between strains and the predominating genotypes within the bioserotype have limited the benefit of these detection methods in epidemiological studies. This method could provide a means of discriminating *Y. enterocolitica* strains found to be identical with other epidemiological tools. There are considerable difficulties associated with isolating *Y. enterocolitica* from clinical, food, and environmental samples. Conventional culture-dependent methods have several limitations, such as low sensitivity, long incubation time, lack of identification between species, and lack of discrimination between pathogenic and nonpathogenic strains. Using PCR, pathogenic *Y. enterocolitica* can be detected in natural samples rapidly and with high specificity and sensitivity. Recently, several real-time PCR assays for qualitative detection of *Y. enterocolitica* in clinical, food, and environmental samples have been developed. However, to date, the PCR method has been used in only a few studies.

Prevalence of pathogenic *Y. enterocolitica* in pigs has been determined by PCR in some countries; however, epidemiological data about other possible animal reservoirs and from many countries are still missing.

Food has often been suggested to be the main source of yersiniosis, although pathogenic strains have seldom been isolated from food samples. Raw pork products have been widely investigated because of the association between *Y. enterocolitica* and pigs. However, the isolation rates of pathogenic *Y. enterocolitica* have been low, which may be due to the limited sensitivity of the detection methods. Occasionally, pathogenic *Y. enterocolitica* has been detected in vegetables and environmental water; thus, vegetables and untreated water are also potential sources of human yersiniosis. To identify other possible transmission vehicles, different food items should be studied more extensively.

Using genotyping, only a few animal reservoirs of *Y. enterocolitica* infections have been identified. The primary source of pathogenic *Y. enterocolitica* is fattening pigs. A close genetic relationship between pig and human strains of *Y. enterocolitica* has been demonstrated by several DNA-based methods. Human pathogenic *Y. enterocolitica* strains share common genotypes with dog strains, indicating that dogs are a possible source of human yersiniosis. In Great Britain, sheep are suspected of being a potential reservoir of human yersiniosis. Similar AFLP patterns between human and sheep strains reinforce this assumption. Wild rodents have been shown to be an important reservoir of *Y. enterocolitica* O:8 strains in Japan. Indistinguishable genotypes have been found among strains isolated from humans and wild rodents. Tonsils of fattening pigs are an important contamination source in slaughterhouses. *Yersinia*-positive tonsils will easily contaminate the carcass, the offal, and the environment during the slaughtering process. Using PFGE, *Yersinia-*contaminated pork and edible pig offal has proven to be important transmission vehicles of pathogenic *Y. enterocolitica* from the slaughterhouse to the retail level and further to humans. Indirect transmission of pathogenic *Y. enterocolitica *from pets to humans may occur via contaminated pork and offal. Indistinguishable genotypes have been found among strains isolated from humans and environmental water, indicating that untreated water is a possible infection source for human yersiniosis. However, many factors related to the epidemiology of *Y. enterocolitica*, such as sources and transmission routes, remain obscure because of the low sensitivity of detection methods and the predominating genotypes among *Y. enterocolitica* strains.

## Figures and Tables

**Table 1:** Biochemical tests used to biogroup *Y. enterocolitica* strains.

Test	Reaction for biotype^a^					
	1A	1B	2	3	4	5
Lipase activity	+	+	−	−	−	−
Salicin (acid production in 24 h)	+	−	−	−	−	−
Esculin hydrolysis (24 h)	+/−	−	−	−	−	−
Xylose (acid production)	+	+	+	+	−	V
Trehalose (acid production)	+	+	+	+	+	−
Indole production	+	+	V	−	−	−
Ornithine decarboxylase	+	+	+	+	+	+
Voges-Proskauer Test	+	+	+	+	+	+
Pyrazinamidase activity	+	−	−	−	−	−
Sorbose (acid production)	+	+	+	+	+	−
Inositol (acid production)	+	+	+	+	+	+
Nitrate reduction	+	+	+	+	+	−

^a^Positive, negative; /: delayed positive; V: variable.

**Table 2:** Relatioship between biotype, O serotype and pYV carriage of *Y. enterocolitica* (adapted from [17]).

Biotype	Serotype(s)
lA	O:4; O:5; O:6,30; O6,31; O:7,8; O:7,13; O:10; O:14; O:16; O:21; O:22; O:25; O:37; O:41,42; O:46; O:47; O:57; NT^a^
1B	O:4,32^b^; O:8^b^; O:13a,13b; O:16; O:18^b^; O:20^b^; O:21^b^; O:25; O:41,42; NT
2	O:5,27^b^; O:9^b^; O:27
3	O:1,2,3^b^; O:3^b^; O:5,27^b^
4	O:3^b^
5	O:2,3^b^

^a^NT: not typable.

^b^Serotypes which include strains that carry pYV.

**Table 3:** Methods for molecular typing of *Yersinia enterocolitica *isolates.

Typing method*	Typeability	Reproducibility	Discriminatory power	Use	Interpretation
REAP	Variable	Good	Poor	Easy	Easy
REAC	Excellent	Moderate	Moderate	Easy	Difficult
Ribotyping	Excellent	Excellent	Variable	Moderate	Easy
PFGE	Excellent	Excellent	Good	Moderate	Easy
PCR	Excellent	Moderate	Variable	Easy	Moderate
AFLP	Excellent	Good	Good	Moderate	Moderate
DNA sequencing	Excellent	Excellent	Good	Difficult	Moderate

Modified from viridi and Sachdeva [18].

*REAP: restriction endonuclease analysis of plasmid; REAC: restriction endonuclease analysis of chromosome; PFGE: pulsed-field gel electrophoresis; AFLP: amplified frgment length polymorphism.

**Table 4:** Annual incidence of disease caused by foodborne bacterial agents in different countries.

Country	Year	Cases	Incidence (per 100 000 population)
Australia	2000	73	0.6
Austria	1998	94	1.2
Belgium	2000	507	5
Denmark	2001	286	5.3
Finland	2001	728	14
Greece	1998	10	0.1
Japan	2001	4	<0.01
Norway	2001	123	2.8
Spain	1998	425	1.1
Sweden	2001	579	6.5
Switzerland	1998	51	0.7
United Kingdom	2000	27	0.05
United States	2002	164	0.44
New Zealand	2006	487	11.8

**Table 5:** Detection of pathogenic *Yersinia enterocolitica* in natural samples.

Sample	No. of samples	Reference
Clinical		
Pig tonsils	185	Fredriksson-Ahomaa et al. [37]
Pig tonsils	252	Boyapalle et al. [56]
Pig feces	255	Boyapalle et al. [56]
Mesenteric lymph nodes	257	Boyapalle et al. [56]
Food		
Pig tongues	51	Vishnubhatla et al. [55]
Minced pork	255	Fredriksson-Ahomaa and Korkeala [7]
Pig offal	34	
Chicken	43	Fredriksson-Ahomaa and Korkeala [7]
Fish	200	
Lettuce	101	
Pork^a^	300	Johannessen et al. [57]
Pig tongues	157	
Ground pork	100	Vishnubhatla et al. [55]
Ground beef	100	
Tofu	50	Vishnubhatla et al. [55]
Ground pork	350	Vishnubhatla et al. [55]
Chitterling	350	Boyapalle et al. [56]
Animal		
Cattle	46	Wang et al. [19]
Goats	160	Wang et al. [34]
Dogs	100	Wang et al. [34]
Swine	196	Wang et al. [34]
Poultry	68	Wang et al. [34]
Environmental		
Water	105	Sandery et al. [58]
Slaughterhouse	89	Fredriksson-Ahomaa et al. [59]
Flies	7	Wang et al. [34]

^a^Except pig tongues and offal.
